# Arginine-Functional Methacrylic Block Copolymer Nanoparticles: Synthesis, Characterization, and Adsorption onto a Model Planar Substrate

**DOI:** 10.1021/acs.biomac.4c00128

**Published:** 2024-05-02

**Authors:** Hubert Buksa, Edwin C. Johnson, Derek H. H. Chan, Rory J. McBride, George Sanderson, Rebecca M. Corrigan, Steven P. Armes

**Affiliations:** † Dainton Building, Department of Chemistry, University of Sheffield, Brook Hill, Sheffield, South Yorkshire S3 7HF, U.K.; ‡ GEO Specialty Chemicals, Hythe, Southampton, Hampshire SO45 3ZG, U.K.; § School of Biosciences, University of Sheffield, Sheffield, South Yorkshire S10 2TN, U.K.; ∥ The Florey Institute for Host−Pathogen Interactions, University of Sheffield, Sheffield, South Yorkshire S10 2TN, U.K.

## Abstract

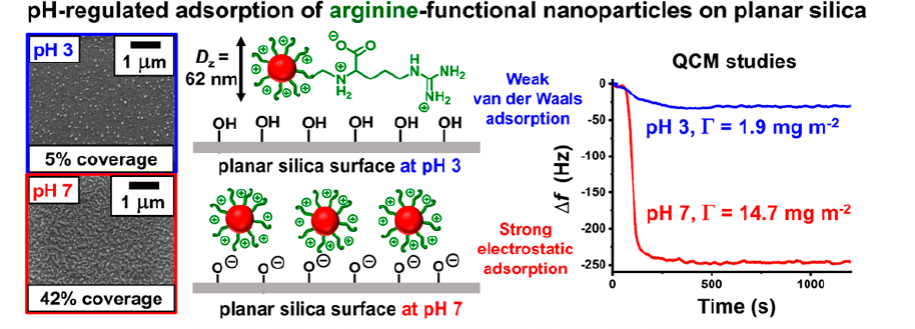

Recently, we reported the synthesis of a hydrophilic aldehyde-functional methacrylic polymer (*Angew. Chem.*, **2021**, *60*, 12032–12037). Herein we demonstrate that such polymers can be reacted with arginine in aqueous solution to produce arginine-functional methacrylic polymers without recourse to protecting group chemistry. Careful control of the solution pH is essential to ensure regioselective imine bond formation; subsequent reductive amination leads to a hydrolytically stable amide linkage. This new protocol was used to prepare a series of arginine-functionalized diblock copolymer nanoparticles of varying size via polymerization-induced self-assembly in aqueous media. Adsorption of these cationic nanoparticles onto silica was monitored using a quartz crystal microbalance. Strong electrostatic adsorption occurred at pH 7 (Γ = 14.7 mg m^–2^), whereas much weaker adsorption occurred at pH 3 (Γ = 1.9 mg m^–2^). These findings were corroborated by electron microscopy, which indicated a surface coverage of 42% at pH 7 but only 5% at pH 3.

## Introduction

Recently, there has been increasing interest in synthetic polymers bearing arginine moieties owing to their potential bioapplications. Arginine-functionalized polymers have been examined as a platform technology for (i) gene or drug delivery and (ii) antimicrobial coatings.^1−4^ More specifically, arginine polymers have been synthesized via Michael-type polyaddition, polycondensation, or radical cross-linking of poly(arginine methacrylate) for use as cell-permeating peptides, polyamide transfection agents, or antimicrobial hydrogels, respectively.^5−7^ In view of the development of multidrug-resistant pathogens such as *Staphylococcus aureus* and *Pseudomonas aeruginosa*,^8^ there has been a concerted effort to prepare new antimicrobial polymers via reversible addition–fragmentation chain transfer (RAFT) polymerization of arginine-mimicking monomers. For example, Xu et al. grew antimicrobial arginine polymer brushes from planar substrates via surface-initiated RAFT polymerization while Perrier and co-workers designed antibacterial diblock copolymers and antifouling star copolymers using arginine-based acrylamides.^4,9,10^ Unfortunately, the requirement for Boc or Fmoc protecting groups and the use of toxic organic solvents such as dichloromethane or dioxane significantly reduces the atom economy and cost-effectiveness of many of the above monomer syntheses. In principle, arginine conjugation to aldehyde-functionalized monomers via imine bond formation offers an attractive alternative route to arginine-functionalized polymers. However, until recently, all suitable aldehyde-functional vinyl monomers have been hydrophobic (e.g., 4-vinylbenzaldehyde) so their statistical copolymerization with a suitable hydrophilic vinyl monomer has been required to produce the desired water-soluble polymer.^11,12^ This approach necessarily limits the degree of aldehyde functionality that can be incorporated into such copolymers.

Over the past decade or so, polymerization-induced self-assembly (PISA) has become an established platform technology for the efficient synthesis of a wide range of block copolymer nano-objects. Many examples of PISA reported in the literature involve RAFT polymerization,^13−29^ and this radical-based chemistry is well-suited for the development of aqueous formulations.^30−45^ For such syntheses, a water-soluble precursor is first prepared via RAFT solution polymerization and then chain-extended using a suitable vinyl monomer to produce an amphiphilic diblock copolymer. Once a critical degree of polymerization (DP) is achieved, the growing second block becomes insoluble and in situ self-assembly occurs to produce nascent diblock copolymer nanoparticles. Depending on the reaction conditions, the initial spherical morphology is either retained or an evolution in morphology occurs to generate highly anisotropic worms, polydisperse vesicles or, in certain cases, lamellae.^46−50^ RAFT polymerization is applicable to many functional vinyl monomers, which has enabled the rational design of a broad range of nanoparticles for various potential applications.^51−56^

Recently, we reported a new synthetic route to controlled-structure poly(amino acid methacrylates).^57^ First, RAFT solution polymerization of a *cis*-diol-functional methacrylic monomer GEO5MA using a suitable dithiobenzoate RAFT agent produced a well-defined PGEO5MA homopolymer (*M*_w_/*M*_n_ < 1.20). Subsequently, selective oxidation of the *cis*-diol groups using NaIO_4_ was conducted in aqueous solution to produce an aldehyde-functional water-soluble precursor, followed by (i) reaction with various amino acids (e.g., glycine, lysine, or cysteine) and (ii) reductive amination to afford the desired poly(amino acid methacrylate). This approach was then extended to include various examples of histidine-functionalized diblock copolymer nano-objects prepared via aqueous PISA^58,59^ and polymer brushes^60^ prepared via atom transfer radical polymerization.^61^

**Scheme 1:**
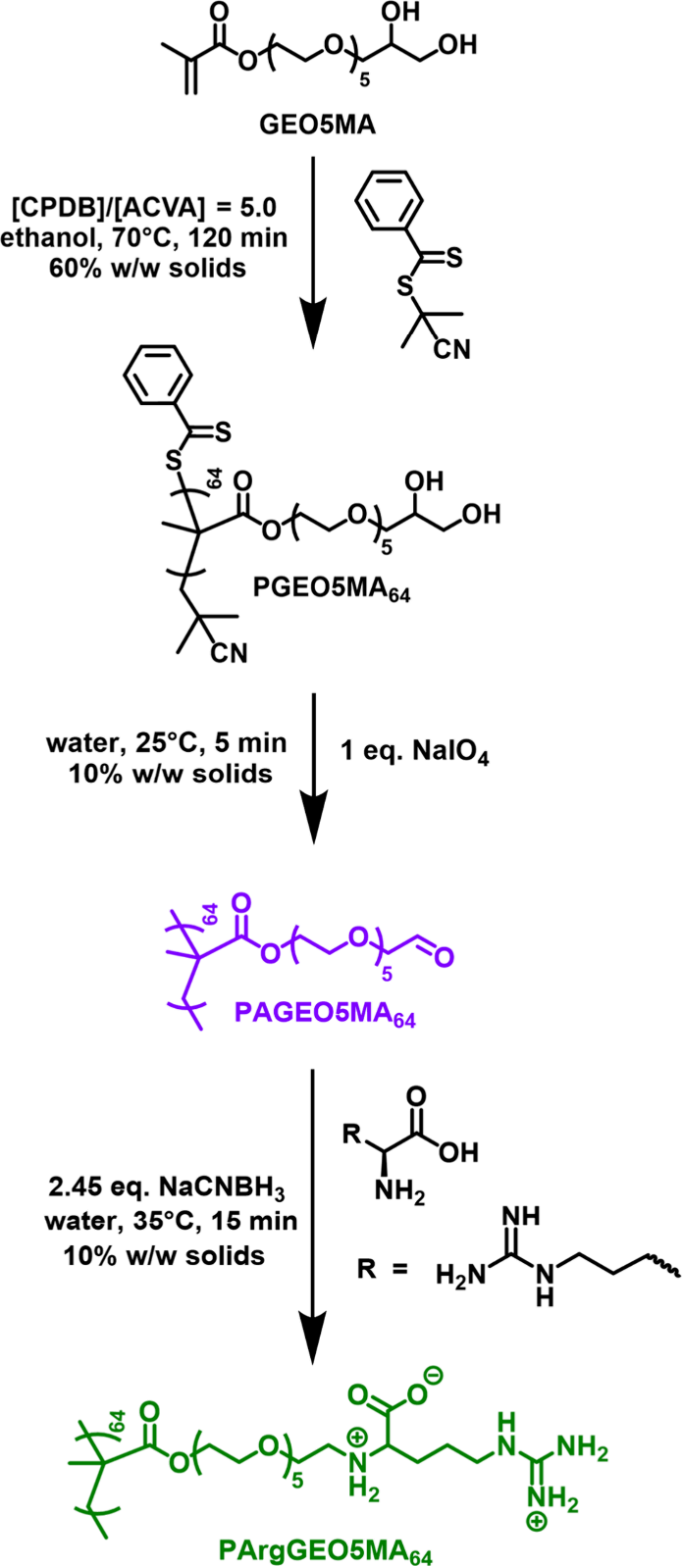
Synthesis of PGEO5MA_64_ via RAFT Solution Polymerization of GEO5MA, Followed by Its Selective Oxidation Using NaIO_4_ in Aqueous Media to Produce the Corresponding Aldehyde-Functional Polymer (PAGEO5MA_64_) Subsequent arginine functionalization at pH 10 produces the target PArgGEO5MA_64_ homopolymer with regioselective control.

**Scheme 2:**
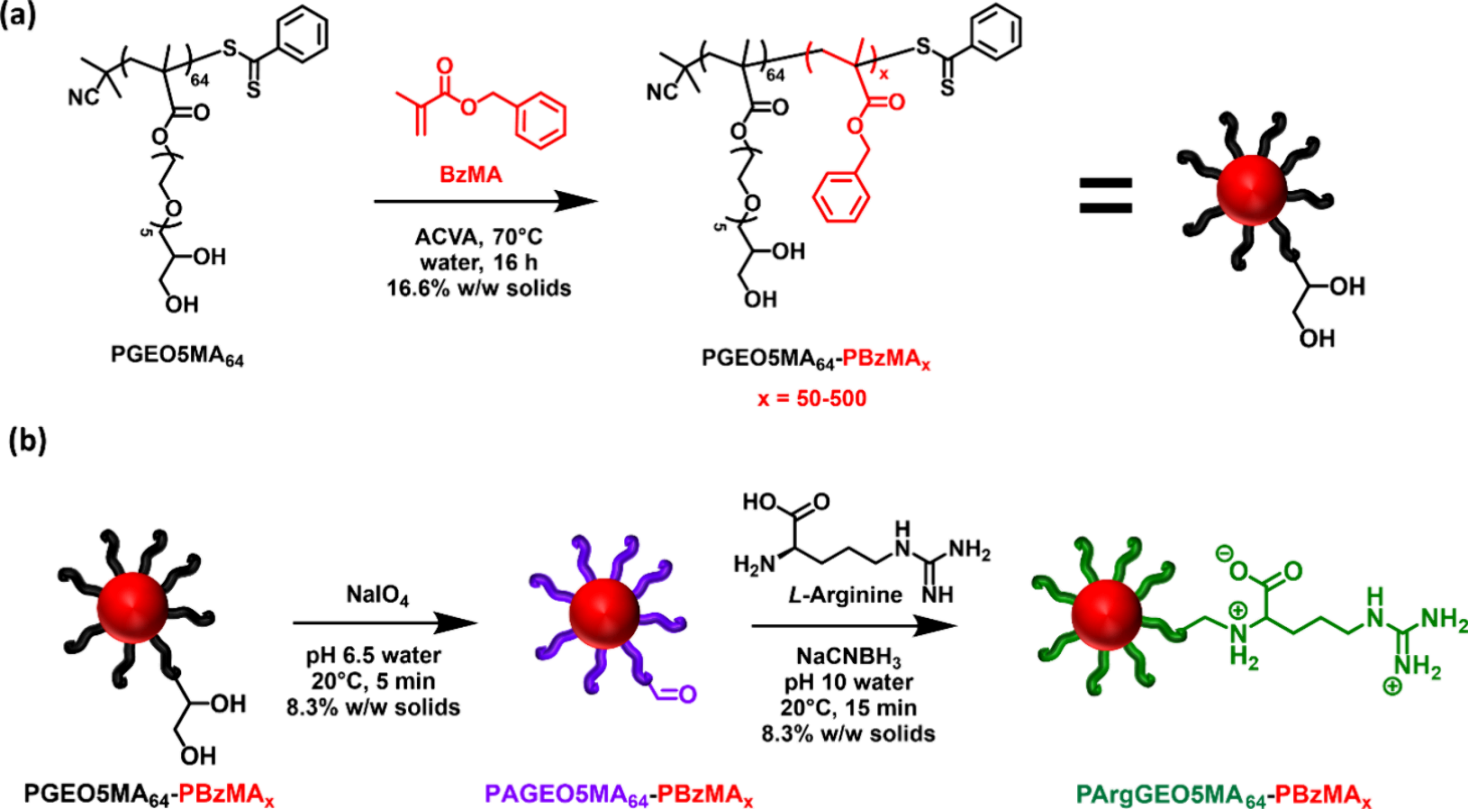
Synthesis of PGEO5MA_64_–PBzMA_*x*_ Nanoparticles via RAFT Aqueous Emulsion Polymerization of Benzyl Methacrylate at 70°C The resulting *cis*-diol functional nanoparticles are first oxidized using NaIO_4_ and then reacted with arginine via Schiff base chemistry, and the subsequent reductive amination using NaCNBH_3_ affords the target arginine-functional nanoparticles.

Herein we exploit the above synthetic strategy to prepare a series of arginine-functionalized diblock copolymer nanoparticles (see Scheme 1). First, GEO5MA is used to prepare a water-soluble PGEO5MA precursor prior to RAFT aqueous dispersion polymerization of benzyl methacrylate to produce sterically stabilized spherical nanoparticles (see Scheme 2). These *cis*-diol-bearing nanoparticles are then reacted with arginine via Schiff base chemistry, followed by reductive amination using NaCNBH_3_. In principle, arginine addition can occur via the primary amine of the amino acid or via the guanidine moiety to produce a binary mixture of isomers. However, we demonstrate that judicious selection of the solution pH ensures regioselectivity during imine bond formation, thus avoiding the protecting group strategy typically employed by others.^1,2,9,10^ The quartz crystal microbalance (QCM) is a well-established surface analytical technique that has been used to study the adsorption of either inorganic or organic particles (including microorganisms) onto various planar substrates, including stainless steel and silica.^54,62−65^ Accordingly, the physical adsorption of such arginine-functionalized nanoparticles on a model planar substrate is examined using QCM in combination with scanning electron microscopy (SEM). In principle, the arginine-functionalized nanoparticles depicted in Scheme 2 constitute an interesting model system for understanding the pH-modulated adsorption of soft nanoparticles onto a hard planar substrate.

## Experimental Section

### Materials

All chemicals were used as received, unless stated otherwise. GEO5MA monomer was kindly donated by GEO Specialty Chemicals (Hythe, UK). 2-Cyano-2-propyl dithiobenzoate (CPDB, > 97%), benzyl methacrylate (BzMA; 96%), 4,4′-azobis(4-cyanopentanoic acid) (ACVA; > 98%), sodium periodate (NaIO_4_, ≥ 99.8%), sodium cyanoborohydride (NaCNBH_3_, 95%), *L*-arginine (≥99.5%), and D_2_O were purchased from Sigma-Aldrich (Gillingham, UK). d_6_-Dimethyl sulfoxide (*d*_6_-DMSO) was purchased from Goss Scientific Instruments Ltd. (Cheshire, UK). Dimethylformamide (DMF, ≥ 99.5%) and ethanol (≥99.5%) were purchased from Fisher Scientific (Loughborough, UK). Deionized water was obtained from an Elga Medica DV25 water purification setup.

### Methods

#### ^1^H NMR Spectroscopy

Spectra were recorded in either D_2_O or *d*_6_-DMSO using a 400 MHz Bruker Avance-400 spectrometer at 298 K with 16 scans being averaged per spectrum.

#### Gel Permeation Chromatography

Aqueous gel permeation chromatography (GPC) was used to determine the number-average molecular weights (*M*_n_) and dispersities (*M*_w_/*M*_n_) for PGEO5MA_64_, PAGEO5MA_64_, and PArgGEO5MA_64_ homopolymers. These polymers were analyzed at 1% w/w using an aqueous buffer eluent containing 0.1 M NaNO_3_, 0.02 M triethylamine, 0.05 M NaHCO_3_, and 0.03% NaN_3_ adjusted to pH 10.0 using 1.0 M NaOH. The GPC setup comprised an Agilent 1260 Infinity instrument equipped with a degasser, pump, a guard column, three columns connected in series (PL-Aquagel Mixed-H, OH-30, and OH-40), and a refractive index detector. The column and detector temperature was set at 35 °C, and the flow rate was 1.0 mL min^–1^. Calibration was achieved using a series of nine near-monodisperse poly(ethylene oxide) standards ranging from 2.1 to 969 kg mol^–1^, and data were analyzed using Agilent GPC/SEC software.

DMF GPC was used to characterize the PGEO5MA_64_ homopolymer and all diblock copolymers. These samples were analyzed at 1.0% w/w using HPLC-grade DMF eluent containing 10 mmol LiBr (Figure S1). The GPC setup comprised an Agilent 1260 Infinity instrument equipped with a degasser, pump, a guard column, two PL-gel 5 μm Mixed-C columns connected in series, and a refractive index detector. Calibration was achieved using a series of 11 near-monodisperse poly(methyl methacrylate) standards ranging from 2.38 to 2200 kg mol^–1^, and data were analyzed using Agilent GPC/SEC software.

#### Dynamic Light Scattering

Dynamic light scattering (DLS) studies were performed using a Malvern Zetasizer Nano-ZS instrument equipped with a 4 mW He–Ne laser (λ = 633 nm) operating at a fixed scattering angle of 173°. Copolymer dispersions were diluted to 0.1% w/w using deionized water prior to light scattering studies at 25 °C, with 2 min being allowed for thermal equilibrium prior to each measurement. The hydrodynamic *z*-average particle diameter was calculated via the Stokes–Einstein equation, which assumes perfectly monodisperse, noninteracting spheres. The polydispersity index is expressed as a standard deviation that indicates the breadth of the particle size distribution, rather than the experimental error.

#### Aqueous Electrophoresis

Zeta potentials were determined using a Malvern Zetasizer Nano ZS instrument equipped with a 4 mW He–Ne laser (λ = 633 nm) operating at a fixed scattering angle of 173°. Diblock copolymer nanoparticle dispersions were diluted to 0.1% w/w with 1 mM KCl as background electrolyte using either dilute HCl or NaOH for pH adjustment as required. Zeta potentials (averaged over three consecutive runs) were calculated via the Henry equation using the Smoluchowski approximation.

#### Transmission Electron Microscopy

Copper/palladium transmission electron microscopy (TEM) grids (Agar Scientific, UK) were coated in-house to yield a thin film of amorphous carbon and were subjected to a glow discharge for 20 s. An aqueous droplet of a copolymer dispersion (7.0 μL, 0.1% w/w) was placed on freshly treated grids for 1 min and then carefully blotted with filter paper to remove excess solution. An aqueous droplet of uranyl formate solution (5.0 μL, 0.75% w/w) was placed on each sample-loaded grid for 1 min and then blotted with filter paper to remove excess stain. This negative staining protocol was required to ensure sufficient electron contrast. Each grid was then carefully dried using a vacuum hose. Imaging was performed at 80 kV using an FEI Tecnai Spirit 2 microscope fitted with an Orius SC1000B camera. Mean nanoparticle diameters were estimated by digital image analysis using *ImageJ* software.

#### QCM Studies

QCM sensors coated with a 50 nm silica overlayer (QSX 303, ~5 MHz fundamental frequency) were purchased from Biolin Scientific (Gothenburg, Sweden). Each sensor was cleaned according to the manufacturer’s instructions. This four-step protocol involved (i) UV/O_3_ treatment for 25 min (Bioforce UV/O_3_ cleaner, 9 mW cm^–2^, λ = 254 nm), (ii) exposure to 2% w/w sodium dodecyl sulfate solution for 30 min, (iii) rinsing with deionized water (iv), drying using a stream of N_2_ gas, and (v) a final UV/O_3_ treatment for 25 min. QCM measurements were performed using an openQCM NEXT instrument (Novatech Srl., Italy) equipped with a temperature-controlled cell connected to a Masterflex Digital Miniflex peristaltic pump (Cole-Parmer Instrument Co Ltd., St Neots, UK). The cleaned substrates were initially equilibrated with deionized water, then exposed to an aqueous dispersion of 1.0% w/w nanoparticles, and finally washed with deionized water to remove any weakly adhering nanoparticles. Measurements were performed at 25 °C using a flow rate of 0.10 mL min^–1^. The mass of adsorbed nanoparticles was calculated using the Sauerbrey equation, which assumes the formation of a rigid thin film and relates the change in resonant frequency (Hz), *Δf*, to the change in adsorbed mass per unit area, *Δm*, via a sensitivity constant *C* (where *C* = −0.177 mg m^–2^ Hz^–1^) and the harmonic number *n*.
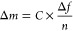
1For the present study, the third harmonic (*n* = 3) was selected to calculate the adsorbed amount (expressed in mg m^–2^ and denoted as Γ) in order to avoid experimental artifacts associated with the fundamental harmonic that can occur if the sample mounting on the sensor is imperfect.^66−68^

#### Scanning Electron Microscopy

After nanoparticle adsorption experiments, selected silica-coated QCM sensors were sputter-coated with a 5 nm layer of gold and SEM images were captured using an Inspect-F instrument operating at an accelerating voltage of 10 kV and a beam current of 200 nA.

### Synthetic Protocols

#### Synthesis of the PGEO5MA_64_ Precursor via RAFT Solution Polymerization in Ethanol

GEO5MA monomer (30.0 g, 0.079 mol), CPDB (0.146 g, 0.66 mmol), ACVA initiator (0.037 g, 0.132 mmol; CPDB/ACVA molar ratio = 5.0), and ethanol (20.0 g) were weighed into a 50-mL round-bottomed flask. This reaction mixture was degassed with N_2_ for 30 min, and the flask was placed in an oil bath set at 70 °C for 120 min. The polymerization was quenched by removing the flask from the oil bath and exposing its contents to air while cooling to 20 °C. ^1^H NMR studies confirmed the GEO5MA conversion to be 53% as judged by the attenuation of the GEO5MA vinyl protons at 5.7–6.1 ppm to the overlapping PGEO5MA and GEO5MA monomer oxymethylene proton signals at 4.1 and 4.3 ppm, respectively. Crude PGEO5MA was purified by precipitation into excess diethyl ether. The precipitate was redissolved in methanol, and the precipitation step was repeated. The PGEO5MA product was redissolved in deionized water, dialyzed for 2 days (with three changes of water per day), and then freeze-dried overnight to produce a red viscous liquid. The mean DP of the purified PGEO5MA precursor was estimated to be 64 via end-group analysis using ^1^H NMR spectroscopy (the integrated aromatic protons assigned to the phenyl end-group derived from the RAFT agent at 7.3–8.0 ppm were compared to the integrated methacrylic backbone protons at 0.80–2.30 ppm).

### Oxidation of PGEO5MA_64_ Homopolymer Using NaIO_4_

PGEO5MA_64_ homopolymer (0.30 g, 12.2 μmol) and 0.70 g of deionized water were weighed into a 15-mL vial and stirred to produce an aqueous solution. Then an aqueous solution of 0.39 M NaIO_4_ (2.0 mL) was added, and the reaction mixture was stirred for 5 min at 25 °C. A NaIO_4_/GEO5MA molar ratio of unity was targeted to ensure full oxidation of the pendent *cis*-diol groups. The degree of oxidation was estimated using ^1^H NMR spectroscopy by comparing the integrated proton signal adjacent to the geminal diol group at 5.1 ppm to that of the oxymethylene proton signal at 4.1 ppm. The resulting 10% w/w aqueous solution of PAGEO5MA was dialyzed against deionized water for two days (with three changes of water per day).

### Reductive Amination of PAGEO5MA_64_ Homopolymer Using Arginine and NaCNBH_3_

*L*-Arginine (49.5 mg, 0.285 mmol) was dissolved in a 10% w/w aqueous solution of PAGEO5MA_64_ homopolymer (1.00 g), and the resulting solution was adjusted to either pH 6 (using 0.1 M HCl) or pH 10 (using 0.1 M NaOH). Then NaCNBH_3_ (43.8 mg, 0.698 mmol) was added, and the reaction mixture was stirred at 35 °C for 15 min before being dialyzed against deionized water for 2 days (with three changes of water per day) to remove impurities and any unreacted reagents. The degree of arginine functionalization was estimated by using ^1^H NMR spectroscopy to monitor the disappearance of the geminal diol proton signal at 5.1 ppm relative to the methacrylic backbone proton signals at 0.8–2.3 ppm. The selectivity of this derivatization was estimated by comparing the −CH_2_–CH_2_–NH– signal intensity at 3.1 ppm from the two aza-methylene protons associated with the amino acid group (see signal *c* in Figure 2) to that at 3.2 ppm from the two aza-methylene protons associated with the guanidine group (see signal *d* in Figure 2).

### Synthesis of PGEO5MA_64_–PBzMA_*x*_ Nanoparticles via RAFT Aqueous Emulsion Polymerization of Benzyl Methacrylate

The following synthesis of PGEO5MA_64_–PBzMA_500_ nanoparticles at 16.6% w/w solids is representative of the general protocol. BzMA monomer (0.31 g, 1.76 mmol), PGEO5MA_64_ precursor (0.086 g, 3.5 μmol; target PBzMA DP = 500), ACVA initiator (0.30 mg, 1.2 μmol; PGEO5MA_64_/ACVA molar ratio = 5.0), and water (2.0 g; targeting 16.6% w/w solids) were weighed into a 15 mL glass vial. The mixture was purged with N_2_ for 15 min, and the vial was placed in an oil bath at 70 °C. After 16 h, the BzMA polymerization was quenched by removing the vial from the bath and exposing the resulting aqueous dispersion to air while cooling to 20 °C. The final BzMA conversion was determined to be more than 99% via ^1^H NMR spectroscopy by monitoring the reduction in intensity of the vinyl proton signals at 5.6–6.2 ppm relative to that of the methacrylic backbone signals at 0.80–2.30 ppm (Figure S2).

### Selective Oxidation of PGEO5MA_64_–PBzMA_500_ Nanoparticles Using NaIO_4_

Oxidation of PGEO5MA_64_–PBzMA_500_ nanoparticles at 8.3% w/w solids was conducted by adding 1.00 g of an aqueous solution of 0.10 M NaIO_4_ to a 15 mL glass vial containing 1.00 g of a 16.6% w/w aqueous dispersion of PGEO5MA_64_–PBzMA_500_ nanoparticles (0.166 g, 1.47 μmol); the resulting reaction mixture was stirred for 5 min at 25 °C. A NaIO_4_/GEO5MA molar ratio of unity was employed to target full oxidation of the pendent *cis*-diol groups. The mean degree of oxidation was estimated using ^1^H NMR spectroscopy in *d*_6_-DMSO (Figure S3). The resulting 8.3% w/w aqueous dispersion of PAGEO5MA_64_–PBzMA_500_ nanoparticles was dialyzed against deionized water for 2 days (with three changes of water per day).

### Reductive Amination of PAGEO5MA_64_–PBzMA_*x*_ Nanoparticles Using Arginine and NaCNBH_3_

The following reductive amination of PAGEO5MA_64_–PBzMA_500_ nanoparticles is representative of the general protocol. *L*-Arginine (8.36 mg, 48.0 μmol; arginine/aldehyde molar ratio = 1.0) was added to a 15 mL glass vial containing 1.00 g of an 8.3% w/w aqueous dispersion of PAGEO5MA_64_–PBzMA_500_ nanoparticles (0.083 g, 0.75 μmol), which was adjusted to pH 10 using 0.1 M NaOH. Excess NaCNBH_3_ (7.39 mg, 118 μmol; 2.45 mol excess) was added, and this reaction mixture was stirred for 15 min at 35 °C. Unfortunately, PArgGEO5MA_64_–PBzMA_500_ proved to be insoluble in common NMR solvents (e.g., *d*_6_-DMSO, CD_3_OD, *d*_5_-pyridine), which precluded analysis using this spectroscopic technique. The resulting 8.3% w/w aqueous dispersion of PArgGEO5MA_64_–PBzMA_500_ nanoparticles was dialyzed against deionized water for 2 days (with three changes of water per day).

## Results and Discussion

### Synthesis of an Arginine-Functionalized Water-Soluble Homopolymer

**Figure 1:**
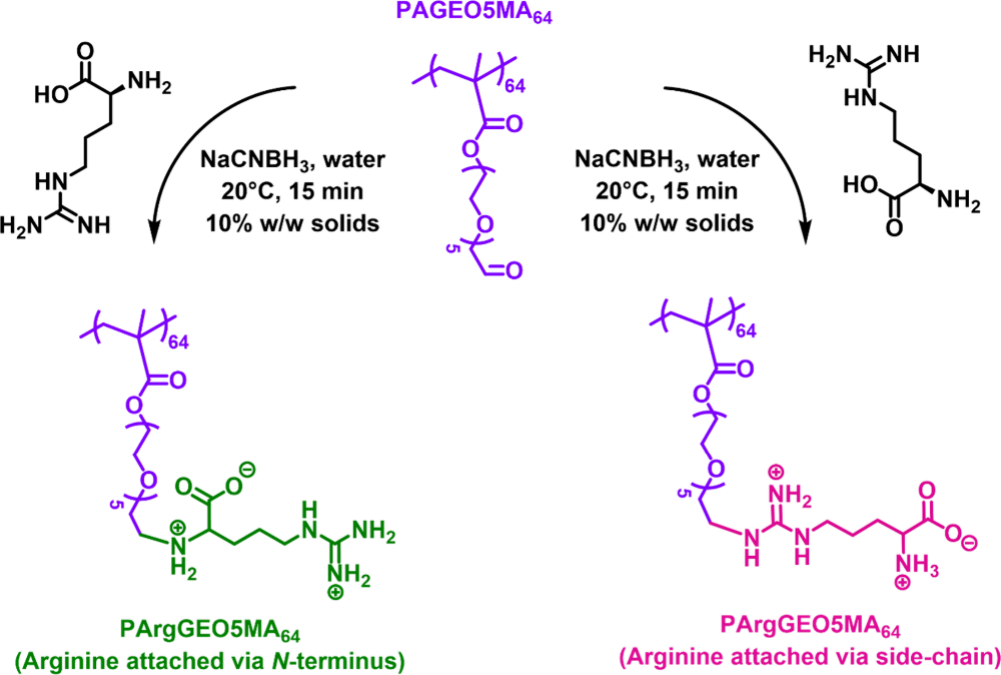
Schematic cartoon depicting the two possible isomers that may be formed when reacting PAGEO5MA_64_ with arginine in the presence of NaCNBH_3_.

We have recently reported the synthesis of the PGEO5MA precursor and its corresponding hydrophilic aldehyde-functional polymer (PAGEO5MA_64_), elsewhere.^57^ In principle, PAGEO5MA_64_ can be derivatized with various amine-functionalized molecules (e.g., amino acids, oligopeptides, proteins or dyes).^57−59^ For example, using arginine should yield an arginine-functionalized polymer, PArgGEO5MA. However, in our initial experiments, we found that reductive amination of PAGEO5MA using arginine at pH 6 yielded a binary mixture of isomers owing to a lack of regioselectivity under such conditions. More specifically, the desired major isomer (arginine attached via the *N*-terminus, see Figure 1) comprised only 79% of the isomeric mixture. This problem arises because both the primary amine and guanidine groups in arginine are protonated at pH 6. Hence, there is insufficient difference between these two potential reactive sites to ensure selectivity.

**Figure 2:**
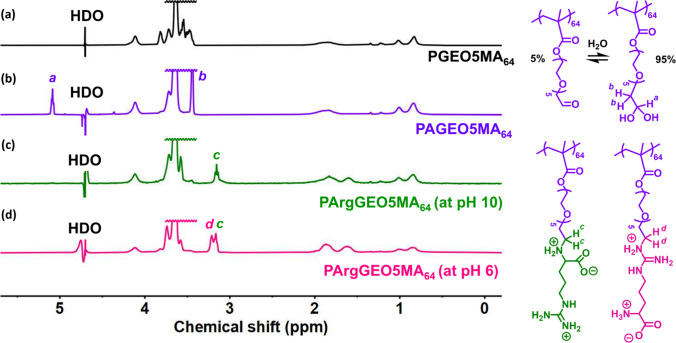
Effect of solution pH on regioselectivity. Partial ^1^H NMR spectra recorded in D_2_O (pH 6; using solvent suppression) for each step during the synthesis of PArgGEO5MA_64_: (a) *cis*-diol functional PGEO5MA_64_ precursor; (b) aldehyde-functional PAGEO5MA_64_; (c) PArgGEO5MA_64_ produced via reductive amination with arginine at pH 10 (regioselectivity under such conditions yields a single isomer); (d) binary mixture of PArgGEO5MA_64_ products obtained via reductive amination with arginine at pH 6 (in this case, poor regioselective control produces two isomers).

Fortunately, the primary amine group within arginine (p*K*_a_ 9.0) exists mainly in its neutral (nonprotonated) form at pH 10, while the guanidine group (p*K*_a_ 13.8) should remain in its protonated form under such conditions.^69^ In principle, this should be sufficient to achieve the desired selectivity. Accordingly, the reductive amination of PAGEO5MA_64_ using arginine was performed at pH 6 and pH 10, and the product(s) of these reactions were analyzed by ^1^H NMR spectroscopy (Figure 2). Periodate oxidation of the PGEO5MA_64_ precursor to form PAGEO5MA_64_ produced two new proton signals associated with the geminal diol group, which is the hydrated form of aldehyde that is obtained in water (Figure 2b). Importantly, reductive amination of PAGEO5MA_64_ with arginine at pH 10 yielded a single product (Figure 2c), as opposed to the binary mixture of isomers obtained at pH 6 (Figure 2d). Clearly, reductive amination of PAGEO5MA_64_ with arginine at pH 10 provides a highly convenient wholly aqueous route to well-defined arginine-functionalized polymers.

**Figure 3:**
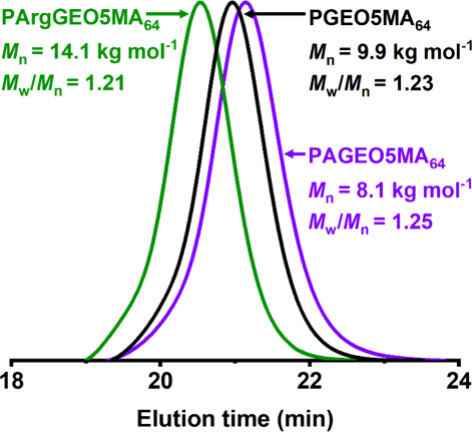
Aqueous GPC curves recorded for the PGEO5MA_64_ precursor prepared via RAFT aqueous solution polymerization of GEO5MA (black curve), the aldehyde-functional PAGEO5MA_64_ homopolymer prepared via selective oxidation of this PGEO5MA_64_ precursor (purple curve), and the PArgGEO5MA_64_ homopolymer obtained via reductive amination after the Schiff base reaction of PAGEO5MA_64_ with arginine at pH 10 (green trace). Apparent *M*_n_ values are expressed relative to a series of near-monodisperse poly(ethylene oxide) calibration standards.

Aqueous GPC was used to characterize the PGEO5MA_64_ precursor, the aldehyde-functionalized PAGEO5MA_64_, and the arginine-functionalized PArgGEO5MA_64_ (Figure 3). Oxidation of PGEO5MA_64_ to PAGEO5MA_64_ involves the loss of formaldehyde, which results in a discernible reduction in *M*_n_ from 9.9 to 8.1 kg mol^–1^. As expected, functionalization of PAGEO5MA_64_ with arginine results in a significantly higher *M*_n_ for PArgGEO5MA_64_. Moreover, the molecular weight distributions obtained for all three polymers are relatively narrow (*M*_w_/*M*_n_ = 1.21–1.25), which indicates that each homopolymer is well-defined and that no side-reactions (e.g., branching or cross-linking) occurred during either oxidation or reductive amination.

### Synthesis and Characterization of Arginine-Functionalized Diblock Copolymer Nanoparticles

**Figure 4:**
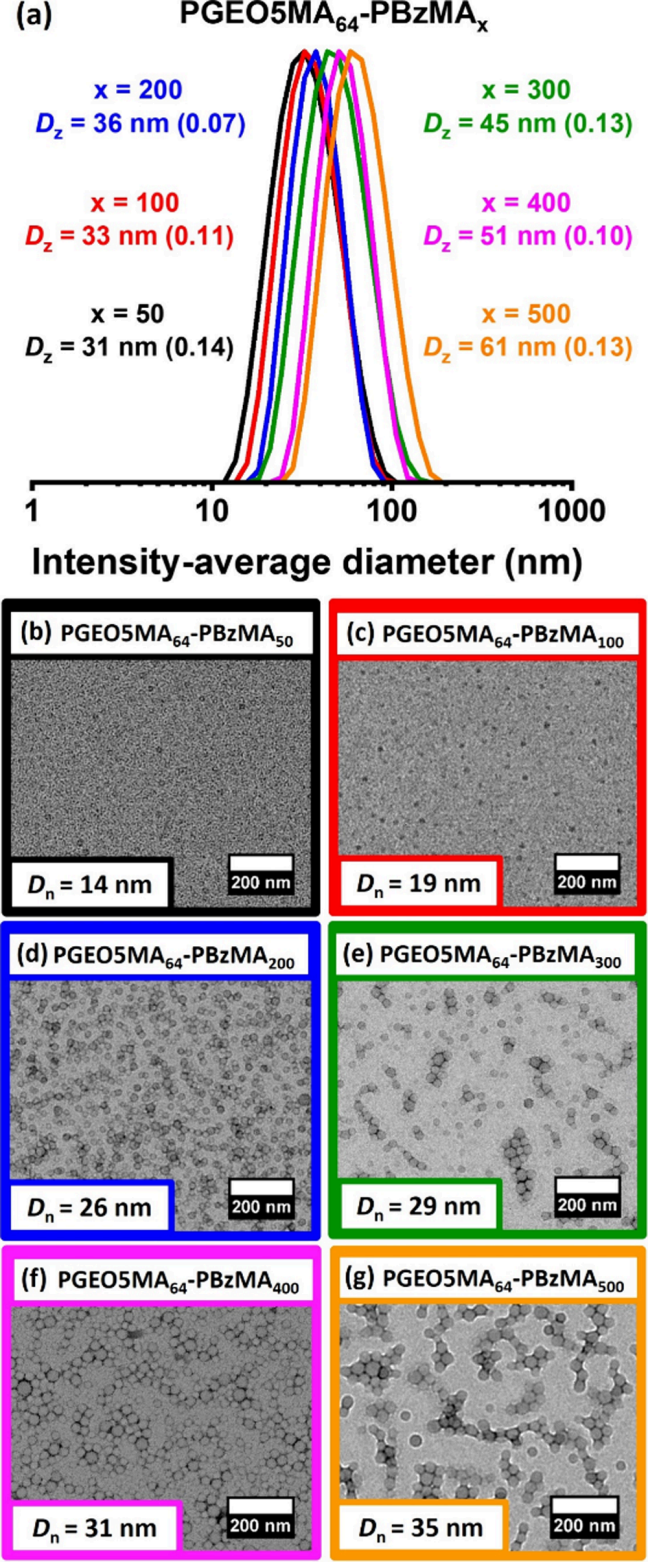
Particle size control by systematic variation of target PBzMA DP. (a) DLS particle size distributions (including *z*-average diameters and DLS polydispersities) and (b–g) corresponding TEM images recorded for a series of six examples of PGEO5MA_64_–PBzMA_*x*_ nanoparticles prepared via RAFT aqueous emulsion polymerization of benzyl methacrylate at 70 °C when targeting a PBzMA DP of 50–500.

Chain extension of this water-soluble dithiobenzoate-capped PGEO5MA_64_ precursor via RAFT aqueous emulsion polymerization of benzyl methacrylate (BzMA) at 70 °C produced a series of PGEO5MA_64_–PBzMA_*x*_ nanoparticles. DMF GPC studies confirmed efficient chain extension and a relatively narrow molecular weight distribution in each case (Figure S1). Systematic variation of the target DP for the core-forming PBzMA_*x*_ block from 50 to 500 produced six aqueous nanoparticle dispersions, with DLS studies indicating *z*-average diameters ranging from 31 to 61 nm (Figure 4a). A plot of such data reveals a monotonic increase in nanoparticle diameter (Figure S4). Similarly, the corresponding TEM images suggest a monotonic increase in the number-average diameter of the nanoparticle cores, *D*_n_, in accordance with prior aqueous PISA formulations (Figure 4b–g).^59,70,71^ In addition, the mean aggregation number, *N*_agg_, or average number of copolymer chains per nanoparticle, was estimated for the smallest and largest nanoparticles. More specifically, the PBzMA core volume, *V*, was calculated from *D*_n_ using *V* = π*D*_n_^3^. Hence the corresponding PBzMA core mass, *m*, is calculated using *m* = ρ·*V*, where the density of PBzMA, ρ, is 1.15 g cm^–3^; dividing *m* by the molar mass of the PBzMA_*x*_ chains gives the mean aggregation number *N*_agg_. Hence PGEO5MA_64_–PBzMA_50_ nanoparticles have an *N*_agg_ of 113, while the PGEO5MA_64_–PBzMA_500_ nanoparticles have an *N*_agg_ of 177.

**Figure 5:**
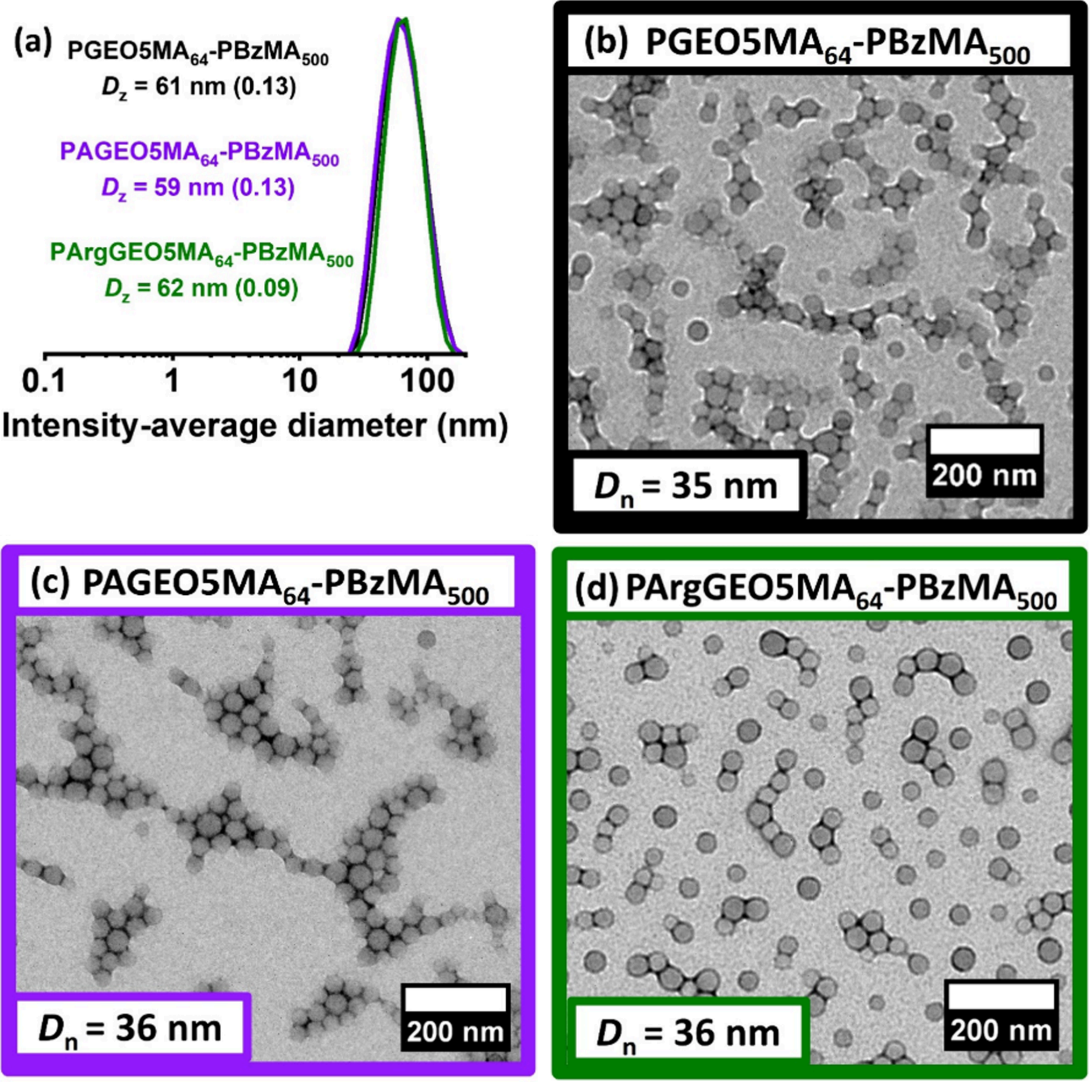
Effect of chemical functionality of the steric stabilizer chains on particle size. (a) DLS particle size distributions (including *z*-average diameters and DLS polydispersities) and corresponding TEM images recorded for (b) *cis*-diol-functionalized PGEO5MA_64_–PBzMA_500_ nanoparticles, (c) aldehyde-functionalized PAGEO5MA_64_–PBzMA_500_ nanoparticles, and (d) arginine-functionalized PArgGEO5MA_64_–PBzMA_500_ nanoparticles.

The largest PGEO5MA_64_–PBzMA_500_ nanoparticles were selected for subsequent derivatization to aid their visualization after adsorption. Accordingly, NaIO_4_ oxidation yielded the corresponding aldehyde-functional PAGEO5MA_64_–PBzMA_500_ nanoparticles, which were subsequently derivatized with arginine via reductive amination at pH 10 to yield cationic PArgGEO5MA_64_–PBzMA_500_ nanoparticles. DLS and TEM studies of the corresponding nanoparticles confirmed that their morphology was not adversely affected during each derivatization (see Figure 5).

**Figure 6:**
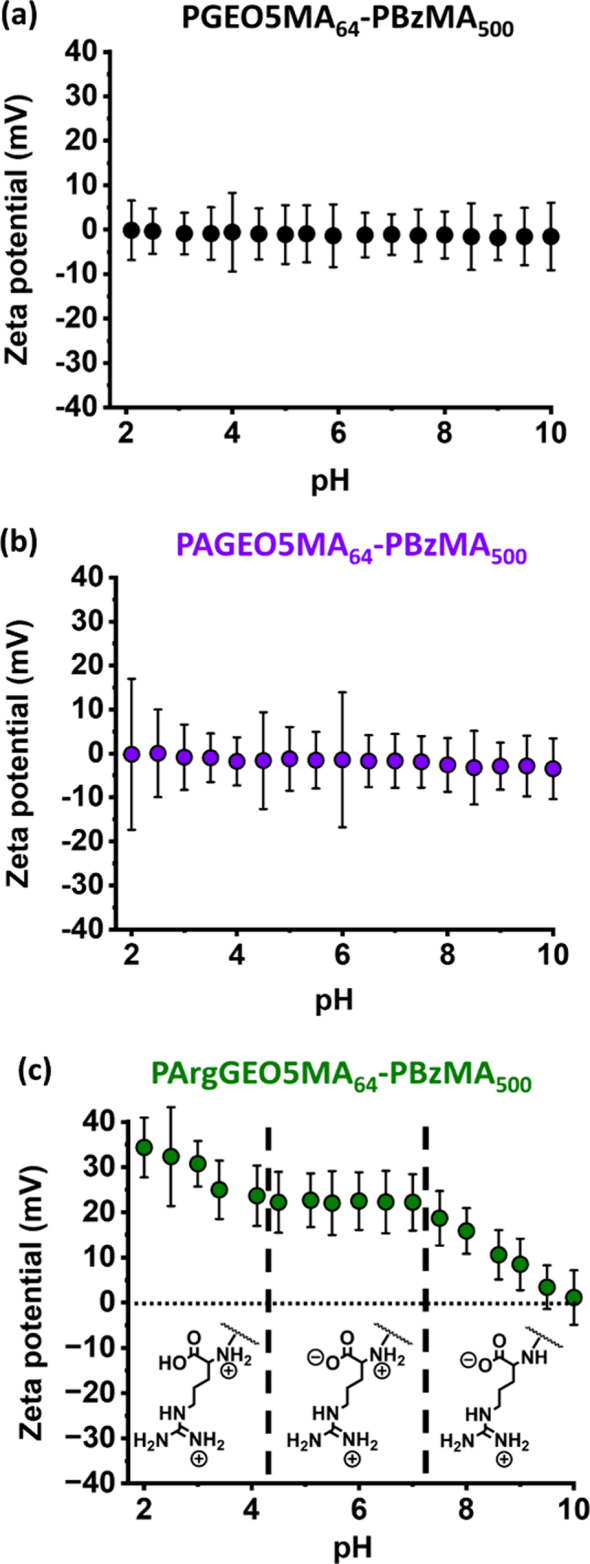
Aqueous electrophoresis data. Zeta potential versus pH curves obtained in the presence of 1 mM KCl for: (a) *cis*-diol-functionalized PGEO5MA_64_–PBzMA_500_ nanoparticles; (b) aldehyde-functionalized PAGEO5MA_64_–PBzMA_500_ nanoparticles; (c) arginine-functionalized PArgGEO5MA_64_–PBzMA_500_ nanoparticles. The two vertical dashed lines at pH 4.2 and pH 7.2 correspond to the approximate p*K*_a_ values for the deprotonation of the carboxylic acid and the primary amine of the pendent amino acid group in PArgGEO5MA_64_, respectively.

Aqueous electrophoresis was employed to assess the change in electrophoretic behavior of these nanoparticles during their derivatization. Accordingly, zeta potential versus pH curves were constructed from pH 2 to 10. As expected, the *cis*-diol-functionalized PGEO5MA_64_–PBzMA_500_ precursor nanoparticles and the aldehyde-functional PGEO5MA_64_–PBzMA_500_ nanoparticles remained essentially neutral across the whole pH range (see Figure 6a and 6b, respectively). In contrast, the PArgGEO5MA_64_–PBzMA_500_ nanoparticles exhibit significant cationic character. A zeta potential of around +34 mV is observed at pH 2, which corresponds to the regime in which the pendent primary amine and guanidine groups are both protonated, and the pendent carboxylic acid group is in its neutral (non-ionized) form. A gradual reduction to a plateau value of +22 mV occurs on raising the pH to 4.3, which then remains constant up to pH 7.2. In this second regime, the carboxylic acid group becomes ionized, which lowers the overall cationic surface charge. A further gradual reduction in zeta potential occurs thereafter owing to deprotonation of the pendent primary amine group, with essentially neutral character observed for these nanoparticles at around pH 10 (Figure 6c). Essentially the same zeta potential versus pH curve was obtained for the smaller PArgGEO5MA_64_–PBzMA_50_ nanoparticles (Figure S5).

### Adsorption Studies of Arginine-Functionalized Diblock Copolymer Nanoparticles

**Figure 7:**
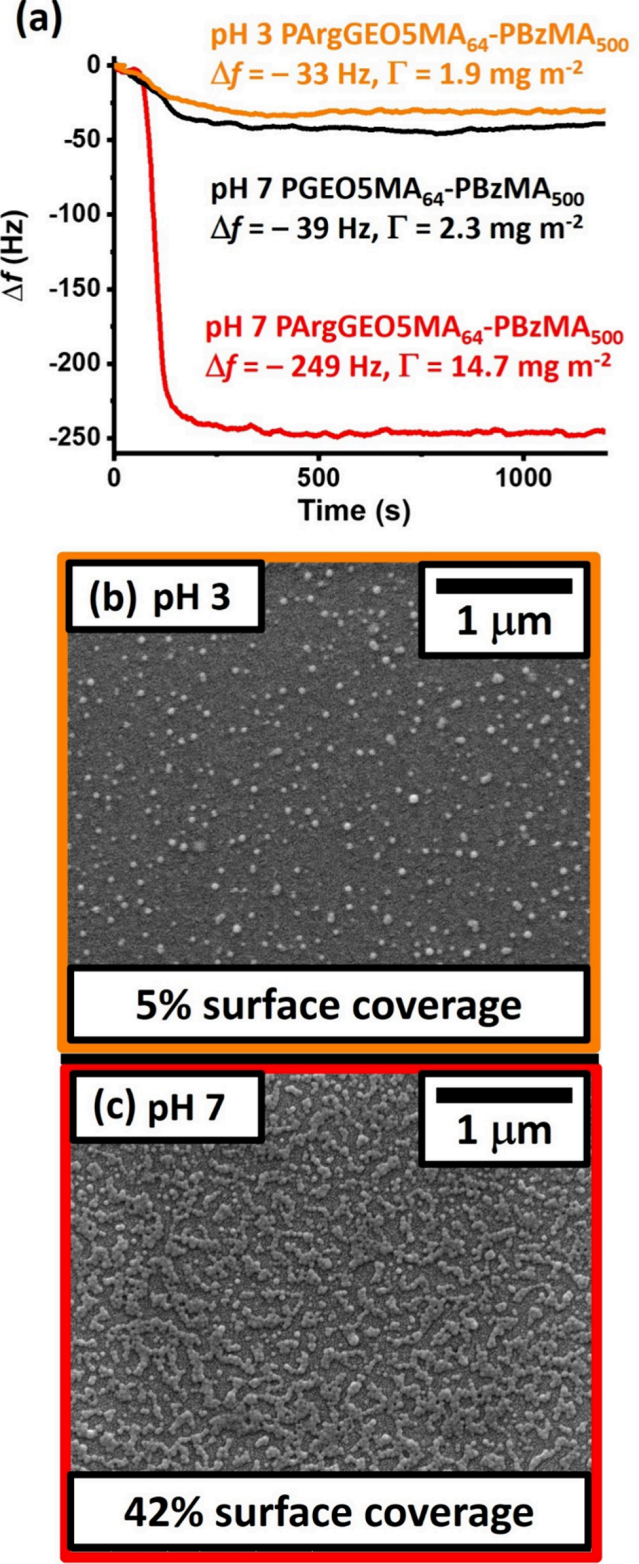
Effect of solution pH and chemical functionality on nanoparticle adsorption at a planar silica substrate. (a) QCM curves recorded during adsorption of neutral *cis*-diol-functionalized PGEO5MA_64_–PBzMA_500_ nanoparticles at pH 7 (black trace); cationic arginine-functionalized PArgGEO5MA_64_–PBzMA_500_ nanoparticles at pH 7 (red trace); and cationic arginine-functionalized PArgGEO5MA_64_–PBzMA_500_ nanoparticles at pH 3 (orange trace). Corresponding SEM images were recorded for arginine-functionalized PArgGEO5MA_64_–PBzMA_500_ nanoparticles adsorbed on the same silica surface at either (b) pH 3 or (c) pH 7. Digital image analysis (*ImageJ* software) indicated surface coverages of 5 and 42%, respectively.

Adsorption of the cationic PArgGEO5MA_64_–PBzMA_500_ nanoparticles onto a model planar substrate (silica) was studied at pH 7 using a QCM (Figure 7a). In such experiments, adsorbed nanoparticles are considered to form a rigid thin film so the Sauerbrey equation is valid. Strong nanoparticle adsorption (Γ = 14.7 mg m^–2^; red curve) is observed at 25 °C. The silica surface is highly anionic at pH 7, which leads to electrostatic adsorption of the cationic nanoparticles. In contrast, despite their greater cationic character (see Figure 6c), nanoparticle adsorption is substantially reduced at pH 3 (Γ = 1.9 mg m^–2^; orange curve). This is because the silica substrate exhibits almost no surface charge under these conditions so nanoparticle adsorption involves only van der Waals interactions. In a control experiment, the neutral PGEO5MA_64_–PBzMA_500_ precursor nanoparticles were also adsorbed onto silica at pH 7. In this case, similarly weak adsorption (Γ = 2.3 mg m^–2^; black curve) was observed, again owing to the absence of any electrostatic attractive interactions. In both cases, the silica sensor was rinsed with deionized water immediately after nanoparticle adsorption, but no discernible change in frequency was observed.

SEM images were recorded for the QCM sensors after performing adsorption experiments using PArgGEO5MA_64_–PBzMA_500_ nanoparticles (Figure 7b). Using digital image analysis (*ImageJ* software), a surface coverage of 42% was calculated for electrostatic adsorption at pH 7 but just 5% surface coverage was estimated for the same nanoparticles adsorbed at pH 3. In summary, these experiments confirm that the extent of adsorption of arginine-functionalized PArgGEO5MA_64_–PBzMA_500_ nanoparticles onto silica is strongly pH-dependent.

**Figure 8:**
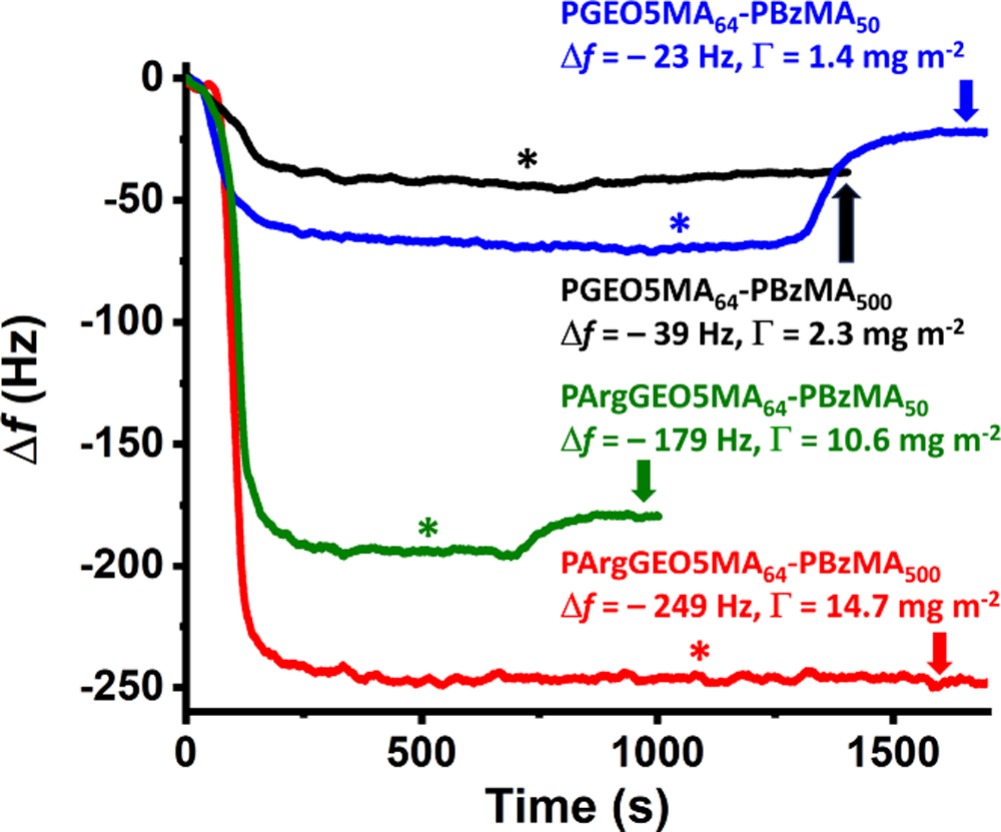
Effect of particle size and chemical functionality on nanoparticle adsorption at a planar silica substrate. QCM curves recorded at pH 7 during adsorption of the smallest (DLS diameter = 31 nm, blue trace) and largest (DLS diameter = 61 nm, black trace) *cis*-diol-functionalized PGEO5MA_64_–PBzMA_*x*_ nanoparticles and the corresponding cationic arginine-functionalized PArgGEO5MA_64_–PBzMA_*x*_ nanoparticles (red and green traces, respectively). The four asterisks indicate the points at which deionized water was introduced to remove any weakly adhering nanoparticles.

Finally, QCM was used to study the adsorption of the smallest [DLS diameter = 31 nm (0.14)] PArgGEO5MA_64_–PBzMA_50_ nanoparticles at pH 7 (Figure 8). As expected, an appreciably lower adsorbed amount (Γ = 10.6 mg m^–2^) was observed compared to that obtained for the 61 nm DLS diameter PArgGEO5MA_64_–PBzMA_500_ nanoparticles (compare green and red curves). Brotherton et al. reported similar observations for the adsorption of sterically stabilized nanoparticles onto stainless steel from aqueous solution.^59^ Moreover, the relatively small PArgGEO5MA_64_–PBzMA_50_ nanoparticles are clearly less strongly adsorbed at the silica surface because a minor fraction (8%) could be removed when rinsing with deionized water. In contrast, no reduction in the adsorbed amount occurs for the larger PArgGEO5MA_64_–PBzMA_500_ nanoparticles. Similar observations were made for the neutral PGEO5MA_64_–PBzMA_50_ and PGEO5MA_64_–PBzMA_500_ nanoparticles: a substantial proportion (68%) of the former could be removed by rinsing, whereas the adsorbed amount obtained for the latter remained essentially unchanged after rinsing (compare blue and black curves). Thus smaller nanoparticles adhere more weakly than larger nanoparticles in the absence of a strong electrostatic attractive interaction between the nanoparticles and the planar substrate. Conversely, introducing such an electrostatic interaction can minimize the partial loss of relatively small nanoparticles during rinsing. In summary, this is an interesting new model system for understanding the effect of particle size and electrostatic attractive forces on the (ir)reversible adsorption of electrosterically stabilized nanoparticles onto oppositely charged planar surfaces.

## Conclusions

We demonstrate that a hydrophilic aldehyde-functional methacrylic polymer can be reacted with arginine in aqueous solution under mild conditions to produce the analogous arginine-functional methacrylic polymer. Importantly, this chemical derivatization can be achieved without recourse to protecting group chemistry. Careful control of the solution pH is essential to ensure regioselectivity for initial imine bond formation; subsequent reductive amination using NaCNBH_3_ leads to a hydrolytically stable amide linkage. This protocol was then utilized to prepare arginine-functionalized diblock copolymer nanoparticles in aqueous media via PISA. Such functionalization did not adversely affect either the nanoparticle size distribution or the molecular weight distribution of the derivatized diblock copolymer chains. Aqueous electrophoresis studies confirmed that these arginine-functionalized nanoparticles exhibit cationic character between pH 2 and 9. A QCM instrument was used to study the adsorption of the resulting cationic nanoparticles onto a planar silica surface. Favorable electrostatic interactions led to strong adsorption at pH 7 (Γ = 14.7 mg m^–2^). In contrast, much weaker adsorption was observed at pH 3 (Γ = 1.9 mg m^–2^) because the silica substrate has almost no anionic surface charge under such conditions. These findings were corroborated by SEM studies, which indicated surface coverages of 42% at pH 7 and 5% at pH 3, respectively. Finally, minimal nanoparticle adsorption was also observed at pH 10 because the nanoparticles are close to their isoelectric point under such conditions.

## Supporting Information Available

DMF GPC curves recorded for the PGEO5MA_64_ precursor and PGEO5MA_64_–PBzMA_*x*_ diblock copolymers; ^1^H NMR spectrum recorded for PGEO5MA_64_–PBzMA_500_ diblock copolymer; and ^1^H NMR spectrum recorded for PAGEO5MA_64_–PBzMA_500_ diblock copolymer (PDF)
