# Supplementary material for: Arginine-Functional Methacrylic Block Copolymer Nanoparticles: Synthesis, Characterization, and Adsorption onto a Model Planar Substrate
Source: Biomacromolecules. 2024 May 2;25(5):2990–3000. doi: 10.1021/acs.biomac.4c00128 (PMC11094727; doi:10.1021/acs.biomac.4c00128)
Supplement: Supplementary file 1 — bm4c00128_si_001.pdf [file bm4c00128_si_001.pdf]

Supporting Information for:

Arginine-functional Methacrylic Block Copolymer Nanoparticles: Synthesis, Characterization and Adsorption onto a Model Planar Substrate

Hubert Buksa[1], Edwin C. Johnson[1], Derek H. H. Chan[1], Rory J. McBride[1], George Sanderson[2], Rebecca M. Corrigan[3,4] and Steven P. Armes*[,1]

1. Dainton Building, Department of Chemistry, University of Sheffield, Brook Hill, Sheffield, South Yorkshire, S3 7HF, UK.

2. GEO Specialty Chemicals, Hythe, Southampton, Hampshire SO45 3ZG, UK.

3. School of Biosciences, University of Sheffield, Sheffield, South Yorkshire, S10 2TN, UK.

4. The Florey Institute for Host-Pathogen Interactions, University of Sheffield, Sheffield, South Yorkshire, S10 2TN, UK.

* Author to whom correspondence should be addressed (s.p.armes@sheffield.ac.uk)

Supporting Figures

DMF GPC traces recorded for a series of PGEO5MA$_{64}$-PBzMA$_{X}$ chains..S2

^{1}H NMR spectrum for PGEO5MA$_{64}$-PBzMA$_{500}$ chains dissolved in d$_{6}$-DMSO S2

^{1}H NMR spectrum for PAGEO5MA$_{64}$-PBzMA$_{500}$ chains dissolved in d$_{6}$-DMSO S3

Z-average diameter vs PBzMA DP plot for six PGEO5MA$_{64}$-PBzMA$_{x}$ formulations S3

Zeta potential vs. pH curve for PArgGEO5MA$_{64}$-PBzMA$_{50}$ nanoparticles..S4

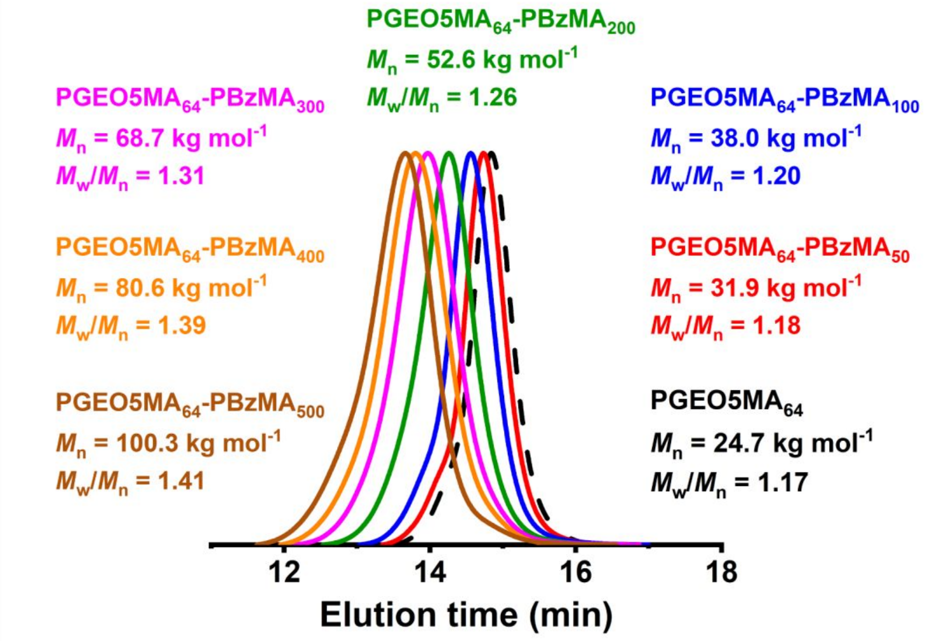

Figure S1. DMF GPC traces recorded using a refractive index detector for a series of PGEO5MA$_{64}$-PBzMA$_x$ nanoparticles prepared via RAFT aqueous emulsion polymerization of benzyl methacrylate at 70°C. M_n values are calculated relative to a series of near-monodisperse poly(methyl methacrylate) calibration standards.

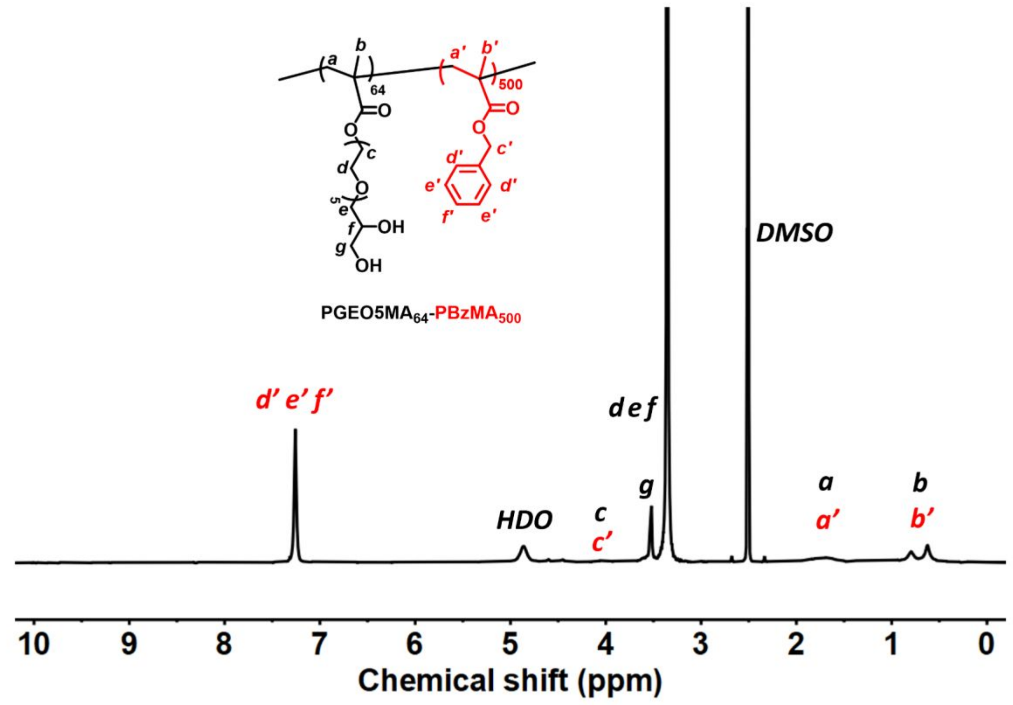

Figure S2. ^{1}H NMR spectrum recorded for PGEO5MA$_{64}$-PBzMA$_{500}$ nanoparticles after their molecular dissolution in d_6-DMSO.

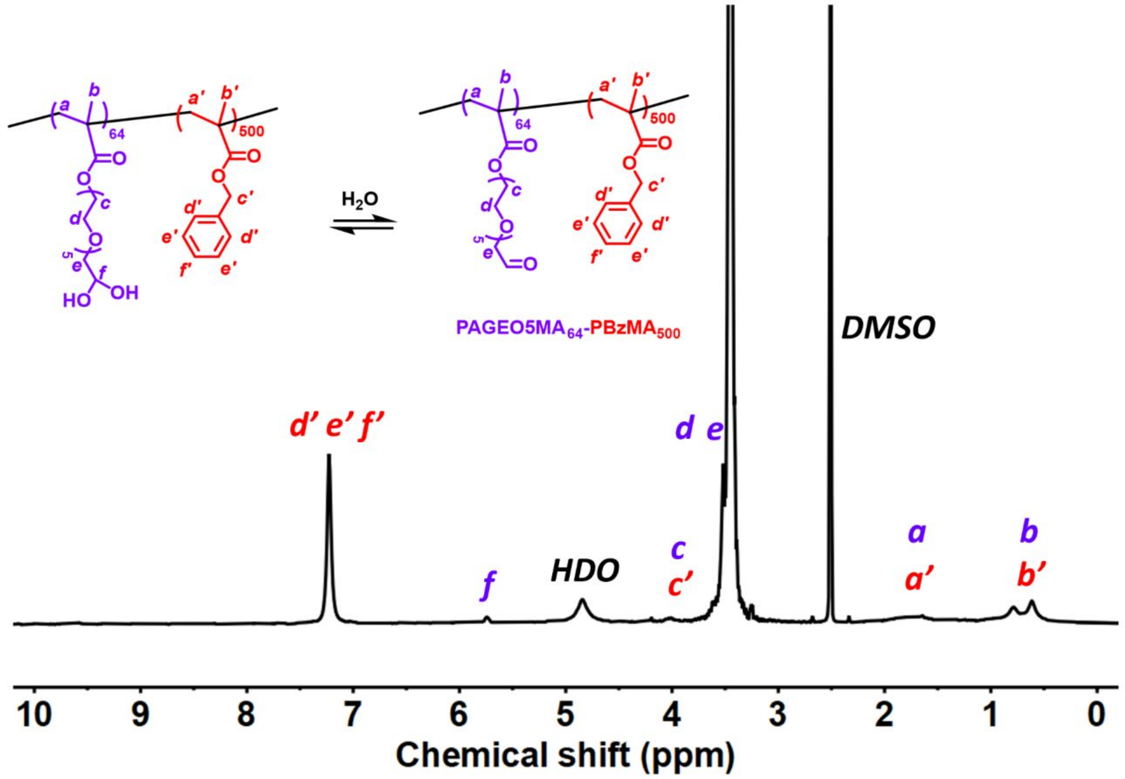

Figure S3. ^{1}H NMR spectrum recorded for PAGEO5MA$_{64}$-PBzMA$_{500}$ nanoparticles after their molecular dissolution in d$_6$-DMSO.

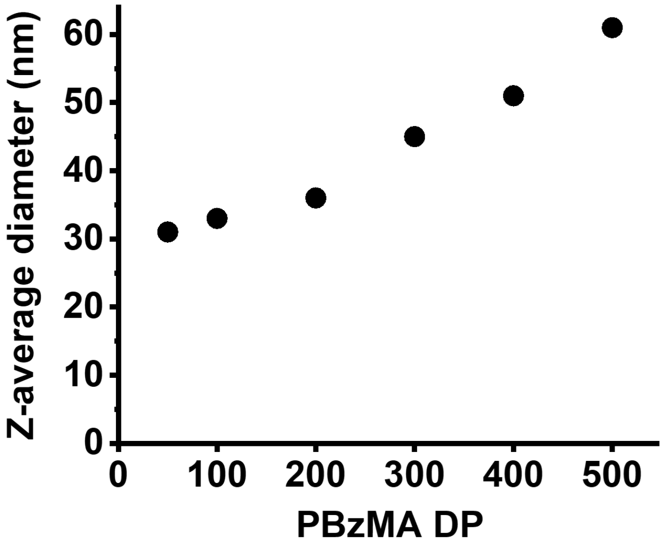

Figure S4. Relationship between the z-average diameter and the PBzMA DP for the series of six PGEO5MA$_{64}$-PBzMA$_x$ formulations discussed in the main manuscript.

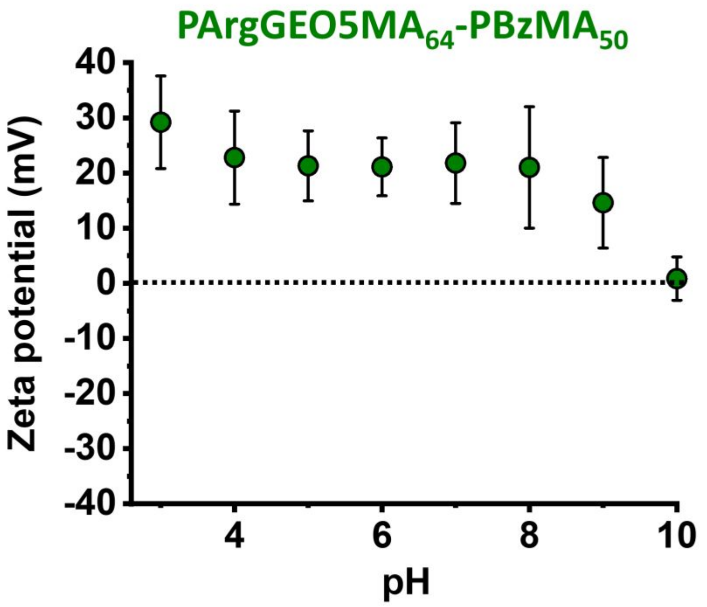

Figure S5. Zeta potential vs. pH curve obtained in the presence of 1 mM KCl for the PArgGEO5MA$_{64}$-PBzMA$_{50}$ nanoparticles.
